# Antimalarial resistance risk in Mozambique detected by a novel quadruplex droplet digital PCR assay

**DOI:** 10.1128/aac.00346-24

**Published:** 2024-05-21

**Authors:** Noah Brown, Clemente da Silva, Caroline Webb, Daniela Matias, Brigite Dias, Beatriz Cancio, Miguel Silva, Ruben Viegas, Crizolgo Salvador, Nordino Chivale, Sonia Luis, Paulo Arnaldo, Julia Zulawinska, Christopher C. Moore, Fatima Nogueira, Jennifer L. Guler

**Affiliations:** 1 Department of Biology, University of Virginia, Charlottesville, Virginia, USA; 2 Global Health and Tropical Medicine, GHTM, Associate Laboratory in Translation and Innovation Towards Global Health, LA-REAL, Instituto de Higiene e Medicina Tropical, IHMT, Universidade NOVA de Lisboa, UNL, Lisbon, Portugal; 3 Instituto Nacional de Saúde, Maputo (INS), Maputo, Mozambique; 4 Hospital Provincial de Matola, Matola, Mozambique; 5 Division of Infectious Disease and International Health, University of Virginia, Charlottesville, Virginia, USA; The Children's Hospital of Philadelphia, Philadelphia, Pennsylvania, USA

**Keywords:** malaria, drug resistance, copy number variation, droplet digital PCR, plasmepsin, multidrug resistance

## Abstract

While the *Plasmodium falciparum* malaria parasite continues to cause severe disease globally, Mozambique is disproportionally represented in malaria case totals. Acquisition of copy number variations (CNVs) in the parasite genome contributes to antimalarial drug resistance through overexpression of drug targets. Of interest, piperaquine resistance is associated with plasmepsin 2 and 3 CNVs (*pfpmp2* and *pfpmp3,* respectively), while CNVs in the multidrug efflux pump, multidrug resistance-1 (*pfmdr1*), increase resistance to amodiaquine and lumefantrine. These antimalarials are partner drugs in artemisinin combination therapies (ACTs) and therefore, CNV detection with accurate and efficient tools is necessary to track ACT resistance risk. Here, we evaluated ~300 clinically derived samples collected from three sites in Mozambique for resistance-associated CNVs. We developed a novel, medium-throughput, quadruplex droplet digital PCR (ddPCR) assay to simultaneously quantify the copy number of *pfpmp3, pfpmp2*, and *pfmdr1* loci in these clinical samples. By using DNA from laboratory parasite lines, we show that this nanodroplet-based method is capable of detecting picogram levels of parasite DNA, which facilitates its application for low yield and human host-contaminated clinical surveillance samples. Following ddPCR and the application of quality control standards, we detected CNVs in 13 of 229 high-quality samples (prevalence of 5.7%). Overall, our study revealed a low number of resistance CNVs present in the parasite population across all three collection sites, including various combinations of *pfmdr1*, *pfpmp2*, and *pfpmp3* CNVs. The potential for future ACT resistance across Mozambique emphasizes the need for continued molecular surveillance across the region.

## INTRODUCTION

Despite decades of research, the *Plasmodium* malaria parasite continues to cause severe disease in humans (1). The global burden of malaria cases reached 247 million and 619,000 deaths in 2021, and the African country of Mozambique accounted for 4.7% of these cases (2). *P. falciparum* is the world’s most deadly malarial parasite and causes infections across this region (3). Research on drug resistance is particularly important since there is no widely employed vaccine and antimalarial drugs are a major tool for control of malaria. Due to Mozambique’s sizable contribution to annual malaria cases, it is crucial to investigate the potential rise of antimalarial resistance in this area.

Artemisinin combination therapies (ACTs) are the most common treatment for *P. falciparum* malaria infections (4). Globally, ACTs are administered with several different partner drugs; in Mozambique, the first-line treatment for uncomplicated malaria is ACT with artemether–lumefantrine (AL), dihydroartemisinin–piperaquine (DHAP), or artesunate–amodiaquine (AS–AQ) (5). Due to reports of low ACT efficacy in Africa (3, 6–9), we aim to determine the risk of AL, AS-AQ, and DHAP resistance emergence in Mozambique.

Mutations in the *pfkelch13* gene are the primary markers of artemisinin partial resistance and are actively being monitored globally. However, the high efficacy of ACTs is in large part due to the inclusion of partner drugs (10, 11). Unfortunately, resistance to the majority of ACT partner drugs has been reported in Africa and Asia (7, 11, 12). Parasite genotypes associated with this resistance predominantly involve copy number variations (CNVs), which increase the transcription of genes encoding for proteins that confer resistance. Key resistance-associated CNVs include *pfmdr*1 (for lumefantrine and amodiaquine) as well as *pfpmp2* and *pfpmp3* genes (for piperaquine) encoding a multidrug efflux pump and digestive proteases, respectively (12–17). It is important to actively monitor these CNVs, particularly in countries like Mozambique where resistance may only just be emerging (18).

In this study, we assessed the presence of resistance-associated CNVs in clinical samples from Mozambique using a novel “quadruplex” droplet digital PCR (ddPCR) assay. This nanodroplet-based method involves end-point PCR and fluorescent probes to amplify and quantify gene copies of interest (19). DdPCR presents an attractive tool for the rapid detection of resistance genotypes in malaria-endemic areas for several reasons. When compared with Sanger sequencing, ddPCR is able to detect minor variants with high accuracy, particularly at lower DNA concentrations (20, 21). When compared to standard quantitative PCR, ddPCR can reduce average costs per sample by 72% by requiring fewer runs to evaluate more loci (22). Furthermore, ddPCR requires less hands-on time per sample due to a lower requirement for replicates and serial dilutions, thus decreasing the chance of technical errors (23). Finally, ddPCR requires sub-nanogram levels of DNA that are easily attainable from minimally invasive collection methods like dried blood spots. While instrument access may limit use in some areas, droplet-based PCR assays are becoming more popular for molecular surveillance (22, 24).

To further extend the benefits of ddPCR, we developed a quadruplex assay, where the three CNV loci (*pfmdr1, pfpmp2,* and *pfpmp3*) and a control gene (*pfhsp70*) are evaluated simultaneously. This approach improves assay efficiency and technical consistency over duplex assays (21). Following optimization, the ddPCR assay proved accurate and sensitive and identified a low but appreciable level of resistance-conferring CNVs across three Mozambique provinces. This study supports the continued need for molecular surveillance across this region to improve the detection of ACT resistance before it becomes more widespread.

## RESULTS

### The optimized quadruplex ddPCR assay is accurate, specific, and sensitive

**Fig 1:**
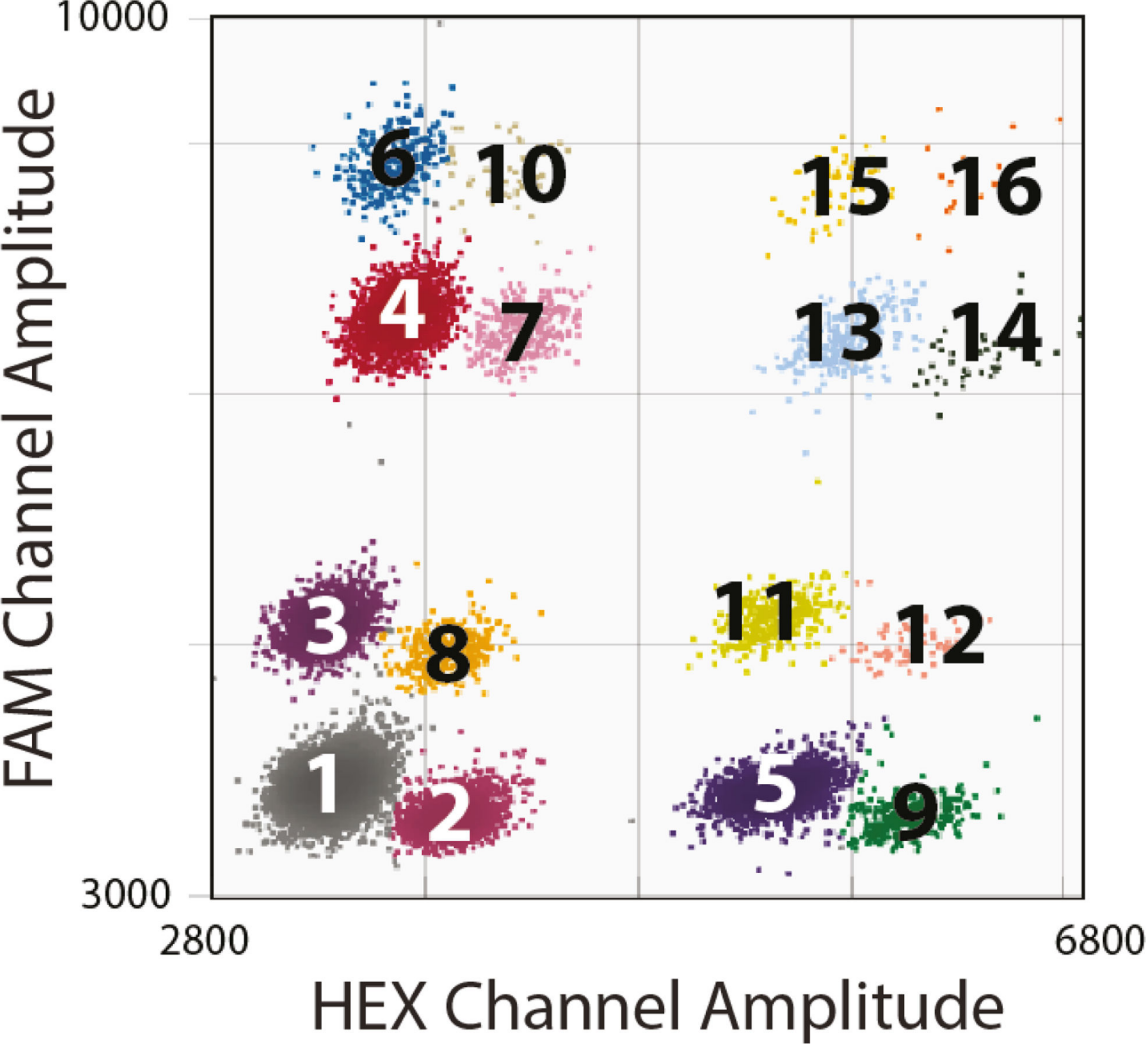
Two-dimensional plot of the quadruplex ddPCR assay. Sixteen clusters of droplets (maximum possible) represent (1): droplets containing no target DNA (negative population), (2): droplets containing at least one copy of *pfhsp70,* (3): droplets containing at least one copy of *pfmdr1,* (4): droplets containing at least one copy of *pfpmp3,* (5): droplets containing at least one copy of *pfpmp2,* (6): droplets with both *pfpmp3* and *pfmdr1,* (7): droplets with both *pfpmp3* and *pfhsp70,* (8): droplets with both *pfmdr1* and *pfhsp70,* (9): droplets with both *pfhsp70* and *pfpmp2,* (10): droplets with *pfmdr1*, *pfhsp70*, and *pfpmp3,* (11): droplets with *pfpmp2* and *pfmdr1,* (12): droplets with *pfhsp70*, *pfmdr1*, and *pfpmp2,* (13): droplets with *pfpmp2* and *pfpmp3,* (14): droplets with *pfhsp70*, *pfpmp2*, and *pfpmp3,* (15): droplets with *pfmdr1*, *pfpmp2*, and *pfpmp3,* (16): and droplets with *pfmdr1*, *pfpmp2*, *pfpmp3*, and *pfhsp70*.

The development of a two-color “quadruplex” assay requires optimization of primer annealing temperature, dilutions, and cycling number (see *Materials and Methods* and Fig. S1 to S3). Copy number determination is based on comparing positive-droplet populations (Fig. 1), which represent the relative proportions of DNA targets in the sample. In our studies, *P. falciparum hsp70* is used as a single copy “reference gene” to determine the copy number of the other “target genes” (i.e., target-CN/reference-CN ≠1 indicates a change in copy number status). With appropriate parasite DNA input (>0.05 ng), the resulting quadruplex assay has 16 distinguishable orthogonal droplet clusters (Fig. 1).

When comparing duplex versus quadruplex assays, we observed no significant difference in copy numbers of *pfmdr1*, *pfpmp2*, and *pfpmp3* using a laboratory parasite line with a known gene copy number (Fig. S4). Additionally, we confirmed that the assays are *P. falciparum*-specific (Fig. S5) and lack interference by human DNA (Fig. S6), which represents the majority of DNA in clinical surveillance samples.

**Fig 2:**
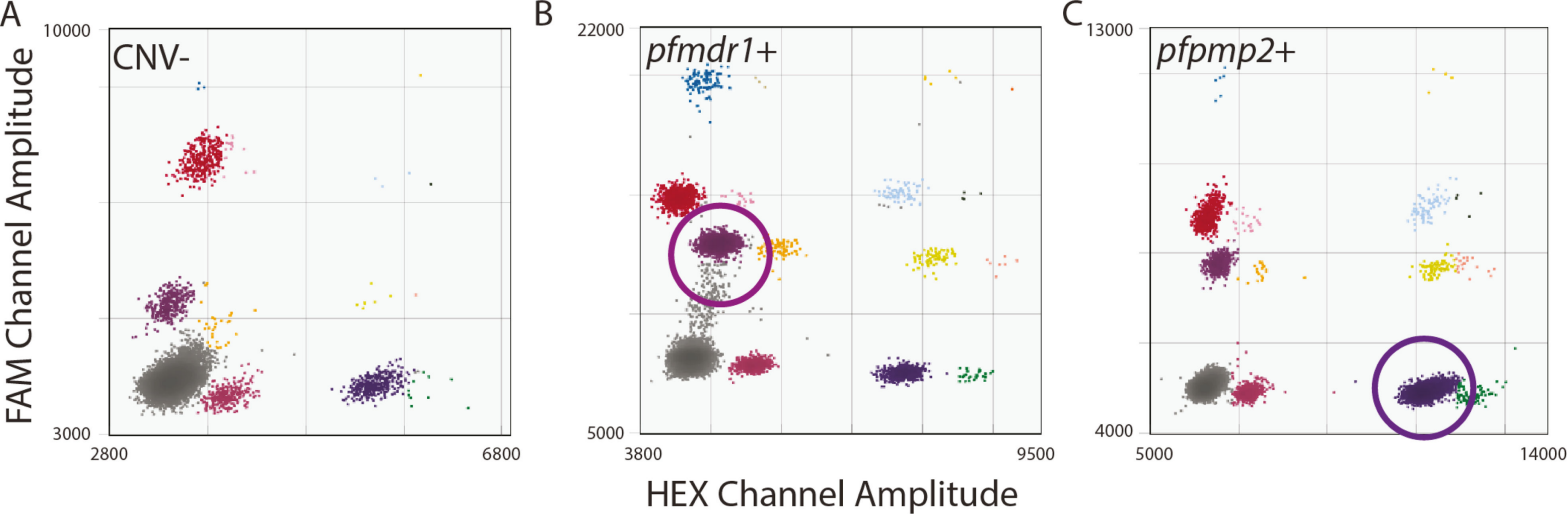
Parasite lines with known CNVs show the accuracy of the quadruplex ddPCR assay. All samples were run at 60 cycles. Clusters of droplets are colored as in Fig. 1. (A) Sample: laboratory-cultured *P. falciparum NF54* DNA with no known CNVs in loci of interest (0.08 ng DNA, 17,739 total droplets, CN: 0.94/1.01/1.09; *pfpmp3, pfpmp2,* and *mdr1,* respectively). Note: this sample is also part of the dilution series represented in Fig S7. (B) Sample: laboratory-cultured *P. falciparum Dd2* DNA with three copies of *pfmdr1* (0.02 ng DNA, 18,400 total droplets, CN: 2.94; *mdr1*). (C) Sample: laboratory-cultured *P. falciparum* PM2GT clone F4 DNA with four copies of *pfpmp2* (0.02 ng DNA, 9,690 total droplets, CN: 4.43; *pfpmp2*).

Assessments that used DNA from laboratory parasite lines also displayed the accuracy and sensitivity of the novel quadruplex assays. As expected, we observed single copies for all ddPCR gene targets for the *NF54* parasite line (Fig. 2A), a three-copy *pfmdr1* CNV for the *Dd2* line (25, 26) (Fig. 2B), and a four-copy *pfpmp2* CNV for the PM2GT clone F4 due to episomal expression (27) (Fig. 2C). When we evaluated the sensitivity of the quadruplex assay across a 5-log range (Fig. S7 and S8A, R_2_ of 0.9766), we detected positive droplets down to 0.08 pg of total parasite DNA (0.00008 ng, Fig. S7A).

**Fig 3:**
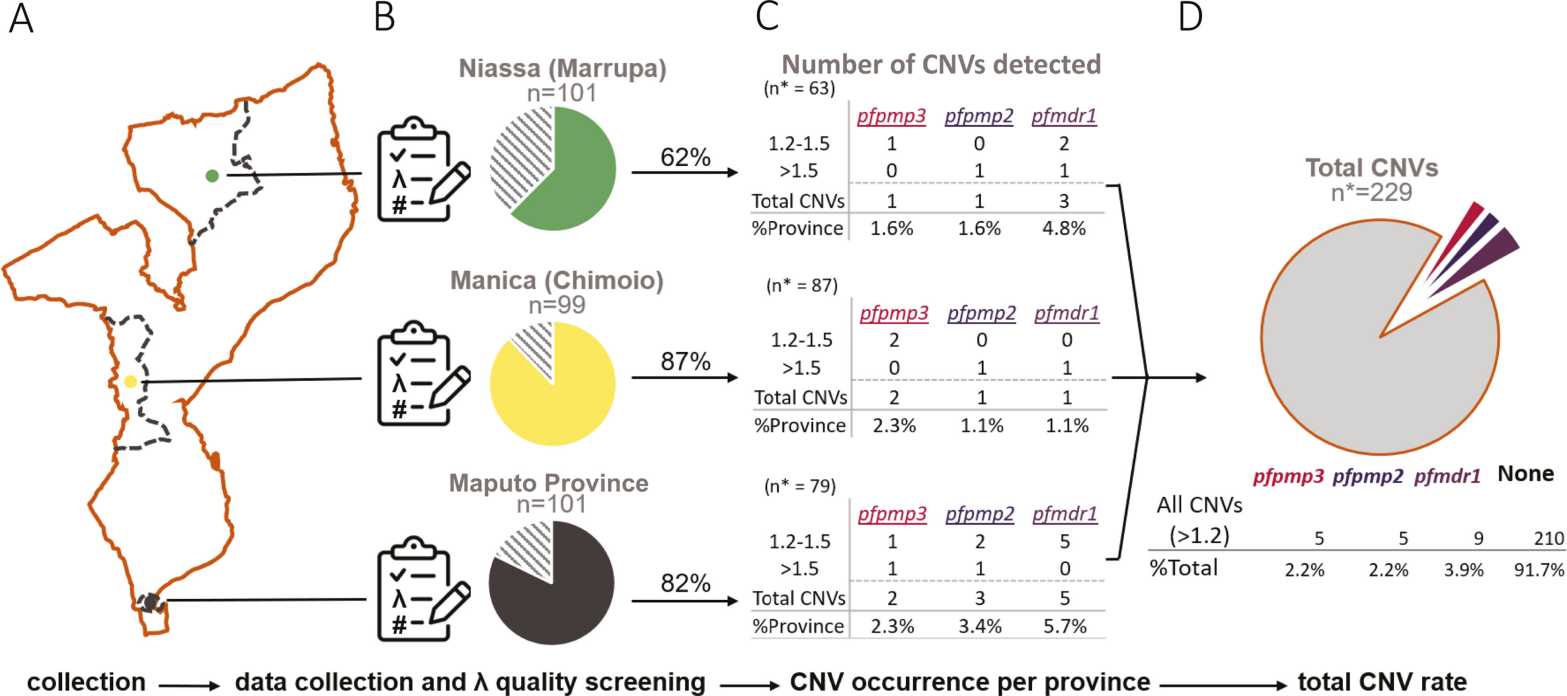
Summary of sample processing and results by province. (A) Depiction of Mozambique sites where samples were collected. Provinces are depicted in dashed lines. The city of collection within the province is depicted with a colored dot. (B) Sample quality control preformed from ddPCR data. Names of the province and city of collection (in parentheses) above pie charts representing the pass rate of quality control using λ (see Materials and Methods); the dashed portion represents failed quality control. *n* = total number of samples collected. (C) Results of ddPCR CNV analysis per-target in each province. n* = total number of samples passing quality screening in each province; 1.2–1.5 represents “potential CNVs”; >1.5 represents “called CNVs”; total CNVs are the sum of the CNVs > 1.2; %Province = Total CNVs/n*. (D) The cumulative total of all CNVs (>1.2) observed from all provinces. n* = combined total samples that passed quality screening.

To explore the impact of genotype mixtures on CNV detection using the novel quadruplex assay, we evaluated mixtures of *P. falciparum* laboratory parasite lines with different genotypes. We were able to detect copy number changes in assays where the CNV+ sample is diluted at various proportions with a CNV- sample (Fig. S9). Based on these data, we can reliably detect CNVs when they are the major genotype (>75%). We also determined that a copy number of >1.5 represents a duplication event in at least 75% of the parasite genomes within the sample. Based on confidence intervals (CIs) from individual experiments, we may be able to detect CNVs at lower levels (i.e., ~50% of the population, Fig. S9A). Therefore, in high-quality samples (low 95% CI), copy numbers between 1.2 and 1.5 were noted as potentially CNV+; this threshold is in line with previous studies that used 1.2 as a cutoff for CNV detection (22).

### Assessment of clinical samples reveals a low level of CNVs across the Mozambique parasite population

Of the 297 clinical *P. falciparum* samples collected from Mozambique (Fig. 3A), we were able to assess 296 samples (99%, Table S1). The majority of samples (229/279, 77%) passed quality screening (λ = 0.005–1.1) due to low 95% CI (Fig. 3B; Table S2; Fig. S8B). When separated by province, we considered 87% of Manica samples, 82% of Maputo samples, and 62% of Niassa samples as high-quality samples (Fig. 3B). Clinical samples with measurable DNA levels fell within the ddPCR positive droplet range of our standards (Fig. S8A), confirming the presence of *P. falciparum* DNA in these samples. During these studies, we detected high-quality clinical samples with as few as 80 positive droplets per target (with a mean of ~16,000 total droplets), which equates to ~0.02 ng of parasite DNA input based on our standard curve (Table S2; Fig. S8A).

Together, we measured a mean copy number for high-quality samples across all loci of 0.95 copies (mean of 0.92, 0.95, and 0.99 for *pfpmp3, pfpmp2,* and *pfmdr1,* respectively; Fig. S10A; Table S2), indicating that the assay did not exhibit locus-specific detection bias. We identified four different high-quality clinical samples that harbored definitive CNVs (>1.5 copies) with six total CNVs (Fig. 3C; Fig. S10A); the samples were spread across the three provinces (one from Maputo, two from Niassa, and one from Manica). Three of the CNVs included the *pfpmp2* gene, two included *pfmdr1*, and one included *pfpmp3*. When we manually inspected ddPCR plots, all of these samples showed high-quality droplet formation and locus detection (Fig. S10B through E). By including samples with copy numbers between 1.2 and 1.5, we identified nine additional high-quality clinical samples across all three provinces that were potentially positive for CNVs in at least one of the three loci (nine for *pfmdr1*, five for *pfpmp3*, and five for *pfpmp2*). Some samples were positive for two CNVs; one sample showed both *pfmdr1* and *pfpmp2* CNVs (Fig. S10C) and four samples with both *pfpmp2* and *pfpmp3* CNVs (Fig. S10D, Table S2).

A portion of samples were rerun to assess the reproducibility of copy number estimates using the quadruplex assay (17 CNV- samples and 13 CNV+ samples). All CNV- samples yielded reproducible calls (Table S1). Despite an additional 18 months of DNA storage, the majority of high-quality samples with potential CNVs (>1.2 copies) also yielded reproducible calls (83% for *pfmdr1*, 67% of *pfpmp3*, and 83% for *pfpmp2*, Table S1).

## DISCUSSION

We identified optimal ddPCR conditions through extensive experimentation including evaluations of cycle number, dilution range, quality control metrics, and potential effects from contaminating DNA from the human host or non-*falciparum* species (Fig. S1 to S7). We established that our novel assay is accurate (Fig. 2; Fig. S4 and S9), specific (Fig. S5 and S6), and sensitive (Fig. S7). We employed metrics to determine sample quality and identified a copy number range for CNV detection in clinical samples. Using this refined approach, we identified a low level of CNVs in clinical parasite populations in Mozambique.

The clinical samples evaluated in our study were prepared from dried blood spots collected after screening for *P. falciparum*. Despite the high sensitivity and accuracy of the ddPCR assay, some samples proved unquantifiable due to low DNA content or quality (Fig. 3B; Table S1). These assay failures were likely due to the age of the purified DNA; in the current study, there was a minimum of 8 months between DNA extraction and ddPCR analysis (maximum of 28 months), which in addition to degradation from freeze–thaw and shipping temperatures (see *Materials and Methods*) could contribute to declining sample quality. Indeed, when samples were repeated ~20 months following the initial runs, we had a failure rate of >50% (Table S1); most of these samples failed due to low positive droplet numbers indicating DNA degradation as the cause.

The *P. falciparum* CNV frequency that we observed in this study was in the range found in previous studies from Mozambique (7, 18). Although we only detected potential CNVs in a total of 13 samples (Table S2), this is appreciative in a small sampling of clinical infections (13/229, 5.7% total; *pfpmp3*: 2.2%; *pfpmp2*: 2.2%; and *pfmdr1*: 3.9%, Fig. 3). For comparison, one study estimated 1.1% (4/350) and 1.4% (5/350) *pfpmp2* and *pfmdr1* CNV prevalence, respectively (18). Another Mozambican study identified multiple copies of *pfpmp2* and *pfmdr1* in 11.3% (8/71) and 16.7% (2/12) of samples, respectively (7).

The incidence of *pfmdr1* CNVs (copy number of >1.2) in our study was approximately twice as common as the *pfpmp2 or pfpmp3* loci (Fig. 3; Fig. S10A). Additionally, of the nine *pfmdr1* CNV+ samples, three samples also harbored *pfpmp2* or *pfpmp3* CNVs (Table S2). Concomitant CNVs may reflect an increase in parasite adaptation to DHAP, which has been directly linked with all three CNVs in genome-wide association studies (8, 16). Interestingly, previous studies from Mozambique did not detect *pfpmp3* CNVs in clinical samples. Although at a very low rate, we detected samples with CNVs (copy number of >1.2) that encompassed both *pfpmp2* and *pfpmp3* genes (3/229, Fig. S10D; Table S2). The report of *pfpmp2/3* co-amplification is consistent with studies from Cambodia, where the CNV region spanned the two neighboring genes (28).

Our data are consistent with the observation that the piperaquine partner drug has not yet been widely used to treat uncomplicated malaria in Mozambique. However, its use during complicated malaria and MDA may select resistant parasite populations. As far as we are aware, this drug has been used for MDA in three Mozambique provinces; DHAP MDA was applied between 2015 and 2017 in the Magude district (29, 30), which is approximately 100 km from the current study health facility (*Hospital provincial da Matola*) in the Maputo province, and in 2021 and 2023 in Cabo Delgado, which is outside of the region of testing in this study. Although a recent study that used the same clinical samples as our study did not detect validated mutations in *pfkelch13* (31), the use of MDA in the region and our identification of a low rate of *pfmdr1* and *pfpmp2/3* CNVs necessitate continued molecular surveillance to better understand genotype dynamics across this region. Fortunately, the prevalence of resistance CNVs in Mozambique remains far below that reported in other countries such as Cambodia (23%) (17), Vietnam (54.3%) (32), and other African countries (average of 21.1% for *pfmdr1* and >30% for *pfpmp2*) (7).

Systematic monitoring of resistance markers from malaria-infected patients using sensitive and accurate assays can assist in the prevention of treatment failure due to resistance (4, 33–35). Our novel quadruplex ddPCR assay is specific for *P. falciparum* loci (Fig. S5), and not suitable for molecular surveillance of *P. ovale* infections, which cause malaria infections in this region of Africa (36, 37). The assay is capable of detecting *P. falciparum* CNVs that are not yet prominent in a parasite population but may be emerging over time (potential CNVs in high-quality samples, >1.2 copies). Therefore this tool, and others capable of detecting minor parasite genotypes, are an important component of malaria resistance surveillance programs; malaria-endemic regions with emerging resistance CNVs can increase malaria prevention efforts to protect from future widespread antimalarial resistance.

## MATERIALS AND METHODS

### Laboratory & control parasites, culturing, and genomic DNA extraction

Laboratory parasite DNA from *P. falciparum* NF54 and Dd2 (MRA1000 and 156, respectively) were used to design and validate assay efficacy before clinical sample use. Parasite culture was carried out as previously (38). Briefly, *P. falciparum* parasites were grown *in vitro* at 37°C at 3% hematocrit (serotype A-positive human erythrocytes) in RPMI 1640 medium (Invitrogen, Waltham, MA, USA) containing 28 mM NaHCO3 and 25 mM HEPES and supplemented with AlbuMAX (TM) II Lipid-Rich BSA (Invitrogen, 11021029) in sterile, sealed flasks, flushed with 5% O_2_, 5% CO_2_, and 90% N_2_ (39, 40). Cultures were maintained with media changes three times each week and sub-cultured as necessary to keep parasitemia below 3%.

DNA from laboratory parasite lines was isolated as previously (41). Parasites were lysed with 0.15% saponin (Sigma Life Science) and washed with 1 x PBS to remove RBC membranes. Then, cells were treated with 200 ug/mL proteinase K (Thermo Fisher Scientific) and 0.1% L-loril sarkosil (Teknova Inc, Hollister, CA, USA) at 37° overnight. DNA was extracted using phenol/chloroform/isoamyl alcohol (25:24:1), pH 7.8–8.1 (Invitrogen), and MaXtract high-density gel tubes (Qiagen) twice, and twice more with only chloroform (Sigma-Aldrich) using standard methods. A final precipitation step with 100% ethanol removed the remaining salts and phenol. The resulting DNA was quantified using the Qubit double-stranded DNA High-Sensitivity kit as per the manufacturer’s recommendations (Invitrogen).

DNA from a laboratory parasite line harboring ~4 copies of *pfpmp2* maintained on the chromosome and episomes was provided by the Michael Klemba (PM2GT clone F4) (27). Clinical samples obtained for this study came from the University of Virginia Medical Center from patients with clinical malaria, as determined by rapid diagnostic tests and peripheral blood smears (see ethical approval above). Blood was obtained from these patients within 24 hours of phlebotomy and used to extract DNA from malaria parasites using the method described above.

### Area of study, clinical isolates, and genomic DNA preparation

Mozambique clinical samples used in this study were obtained from patients admitted in health facilities from three settings, namely, Northern (Hospital distrital de Marrupa in Niassa), Central (Centro de Saúde Eduardo Mondlhane and Centro de Saúde 7 de abril in Manica), and Southern (Hospital provincial da Matola in Maputo) areas of Mozambique, between April and August of 2021, as previously described (31). The choice of Niassa, Manica, and Maputo provinces not only aligns with our scientific goals but also with the objectives of Instituto Nacional de Saúde and National Malaria Control Programme Mozambique, contributing to the systematic mapping of malaria cases at the national level.

A total of 450 participants of all ages with malaria-positive rapid diagnostic test (RDT) were recruited and provided 100 µL of blood samples on filter paper (Whatman FTA cards), after written informed consent. All dried blood spot samples were then stored under −20°C until they were used for genotyping.

Parasite genomic DNA from dried blood spots was extracted using the Chelex method (42), and DNA was stored at −20°C. Real-time PCR for *P. falciparum* confirmation, targeting the 18S rRNA gene, was conducted as described in da Silva et al. (31). *P. falciparum* positive DNA samples (*n* = 297) from three provinces (Maputo *n* = 97, Niassa *n* = 100, and Manica *n* = 100) were assessed at the University of Virginia (shipped at room temperature, stored at −20°C). Clinical isolate DNA concentration ranged from 0.1 to 2.0ng/μL with an average concentration of ~0.4 ng/µL. Samples were coded at collection, and location details were blinded during experimentation.

### Droplet digital PCR quadruplex assays and CNV analysis

To achieve optimal primer/probe concentrations, the two-color quadruplex assay was developed as two individual duplex assays on either fluorescence channel and then merged into a single quadruplex assay with small adjustments (Table 1: primer and probe concentrations). Because the ddPCR droplet-analyzer used in this study possesses two fluorescence channels (FAM and HEX), quadruplexing requires an intentional decrease in the fluorescence amplitude of one of the assays on each channel to distinguish the populations. The amplitude of these droplet clusters can be affected by several factors including the age of the primers and probes, purity of the input DNA, and interexperimental variables like UV exposure and droplet generation variance (43). Generally, when shifts in amplitude occur from interexperimental factors, they affect all clusters equally, allowing for accurate assignment of droplet clusters.

**TABLE 1:** Optimized quadruplex assay reaction primer and probe concentrations^*a*^

Target	Probe concentration (nm)	Fluorophore	Primer concentration (nm)
***pfpmp2^a^***	125	HEX	2,500
*pfhsp70^b^*	125	HEX	800
***pfpmp3^a^***	50	FAM	400
*pfmdr1*	125	FAM	600

^*a*^ Bold targets indicate assays with higher fluorescence amplitudes on ddPCR plots.

^*b*^ Used as a single-copy reference gene.

Primer and probe sequences (Table 2) were BLASTed against Pf3D7 and human genomes to confirm the absence of potential secondary targets and assessed for potential dimer formation using the IDT OligoAnalyzer tool. The specificity for *Plasmodium* genes was also confirmed by the lack of amplification of human genomic DNA (Fig. S6). Optimal assay temperature was assessed by performing assays in duplex over a thermal gradient of annealing/extension temperatures and determining the condition with the highest average amplitude Fig. S1).

**TABLE 2:** Primer and probe sequences for the quadruplex ddPCR reaction

Target	Forward primer	Reverse primer	Probe
*pfpmp2* ^*a*^	5′-ATGGTGATGCAGAAGTTGGA-3′	5′-AACATCCTGCAGTTGTACATTTAAC-3′	5'-/5HEX/-CAGGATCTGCTAATTTATGGGTCCCA/3IABkFQ/-3'
*pfhsp70* ^*b*^	5′-TGCTGTCATTACCGTTCCAG-3′	5′-AGCAGCTGCAGTAGGTTCATT-3′	5'-/5HEX/AGATGCTGGTACAATTGCAGGA/IABkFQ/-3'
*pfpmp3* ^*a*^	5′-CCACTTGTGGTAACACGAAATTA-3′	5′-TGGTTCAAGGTATTGTTTAGGTTC-3′	5'-/56FAM/CCAACACTCGAATATCGTTCACCAA/IABkFQ/-3'
*pfmdr1* ^*b*^	5′-TGCCCACAGAATTGCATCTA-3′	5′-TCGTGTGTTCCATGTGACTG-3′	5'-/56FAM/ACCCTGATCGAAATGGAACCT/IABkFQ/-3'

^*a*^ These primers and probes were previously reported in (28).

^*b*^ These primers and probes were previously described in (44).

Purified bulk DNA (~10 ng/µL) from laboratory parasite lines was digested with BamHI (New England BioLabs) for 12 hours at 37°C and then diluted to 0.004 ng/µL for input into ddPCR assays (total of 0.02 ng). Extracted DNA (5 µL) from clinical samples was directly digested with BamHI and diluted at a ratio of 1:2-1:10 into the ddPCR assay (Table S1; Fig. S2). Some samples were run undiluted to account for low parasite DNA amount. The BamHI restriction enzyme was chosen specifically due to a predicted restriction site located between the tandem *pfpmp2* and *pfpmp3* genes, while generating similar lengths for all target-containing DNA fragments including *pfhsp70 and pfmdr1* (10–60 kb).

Fresh primer/probe master mixes were prepared for each assay at 10x final concentrations (Tables 1 and 2). Each 23 µL ddPCR reaction contained 5 µL of restriction-digested DNA (diluted as mentioned above), 1:10 dilution of each 10 x primer/probe mix (four sets), and 1:2 dilution of ddPCR Supermix for probes (Bio-Rad Laboratories). Reactions underwent droplet generation using a QX200 Droplet Generator with droplet generation oil for probes (Bio-Rad Laboratories) and subsequent PCR using a C1000 Thermal Cycler (Bio-Rad Laboratories). Thermal cycling conditions are as follows: 10 min at 95°C initial denaturation step, 1 min at 95°C second denaturation step, and 2 min at 58°C annealing and extension step (ramp rate of 1°C per second), the second denaturation step and the annealing/extension step repeated 60 times, and then 10 min at 98°C to halt the reaction. This longer cycling program (60 cycles versus the standard 40 cycles) increased ddPCR assay success for lower-quality clinical samples (Fig. S3). Samples were kept at 4°C until use for analysis (no longer than 24 hours).

After amplification, probe fluorescence was read using a QX200 Droplet Reader (Bio-Rad Laboratories) and analyzed using QuantaSoft Software version 1.7.4 (Bio-Rad Laboratories). Populations were manually defined as instructed by the manufacturer using QuantaSoft software. Samples were only considered valid if a minimum of 10,000 total droplets were observed. Poisson confidence intervals provided by the software were used to assess sample quality.

Samples were screened for quality using the lambda (λ) value in accordance with the Minimum Information for Publication of Quantitative Digital PCR Experiments (22, 45, 46), where λ = ln(#accepted droplets/#negative droplets). Samples passed quality screening if λ was between 0.005 and 1.1. This threshold was chosen as it represented high-quality clinical samples, where the difference between the maximal 95% CI value and the determined copy number was less than 30% of the copy number value ([(MaxCI – CopyNumber) / CopyNumber] <30%, Fig. S11, blue box). Furthermore, during this calculation, if the accuracy (defined as: %100 * |1 – CopyNumber|) deviates from the expected value of “1”, it is either a product of insufficient quantification or potential CNV signal (Fig. S11, green box).

CNVs were identified with QuantaSoft Software. Each droplet was automatically designated as positive or negative for template DNA through the detection of the fluorescence signal after amplification (Fig. 1 ). The relative abundance of a single-copy reference (*pfhsp-70*, PF3D7_0818900) compared to a target amplicon was used to detect CNVs (*pfmdr1*, PF3D7_0523000; *pfpmp2*, PF3D7_1408000; *pfpmp3*, PF3D7_1408100, e.g., a target/reference abundance of 2:1 indicates a duplication of the target). Samples that passed quality screening (see above) and possessed a ratio ≥1.5 target/reference were considered positive for CNVs. Samples with copy numbers between 1.2 and 1.5 were manually inspected and considered potentially positive if samples were high quality and droplet populations were well-resolved.

### High-resolution melting for evaluating *Plasmodium* species

High-resolution melt (HRM) assays were used to validate the *Plasmodium* species of samples used to test the specificity of the quadruplex assay (47). Plasmid controls containing partial species-specific 18S rRNA gene sequences were obtained through BEI Resources (Manassas, VA, USA) (*P. falciparum* (MRA-177, lot: 70042650), *P. vivax* (MRA-178, lot: 70041752), *P. malariae* (MRA-179, lot: 70051095), and *P. ovale wallikeri* (MRA-180, lot: 70043212). Each 25 µL reaction contained 2 x HRM PCR Master Mix (Type-it HRM PCR Kit, Qiagen), 700 nM of primers targeting the multicopy 18S rRNA gene: 5′-GTT CCT CTA AGA AGC TTT-3′ and 5′-TAA CGA ACG AGA TCT TAA-3′ (48) (IDT, USA), ~10 ng of the template DNA, and RNase-free water. The PCR was performed with the following cycling conditions: 95°C for 5 min, 45 cycles of 95°C for 10 s, 55°C for 20 s, followed by an HRM ramp from 65°C to 95°C, with an increase of 0.1°C every 2 s on the Rotor-Gene Q real-time PCR instrument with a 72-well rotor (Qiagen). Rotor-Gene Q software (version 2.3.5, build 1; Qiagen) was used to plot the change in fluorescence versus temperature (dF/T), which allowed us to compare the HRM peaks of the clinical samples and plasmid controls. Multiple peaks are diagnostic of genomic DNA samples (from clinical or laboratory sources) from specific *Plasmodium* species (47). These peaks are due to amplification of distinct 18S rRNA gene copies present in the genomes (# of total 18 s rRNA genes in genome/# of 18 s rRNA genes with primer homology: *P. falciparum* 5/5; *P. vivax* 3/2; and *P. ovale wallikeri* 2/2).

## SUPPLEMENTAL MATERIAL

Supplemental figures aac.00346-24-s0001.docx Figures S1 to S11.

Table S1 aac.00346-24-s0002.xlsx Clinical sample ddPCR assessment including dilutions and repeats.

Table S2 aac.00346-24-s0003.xlsx Clinical sample CNV calls and quality metrics.

ASM does not own the copyrights to Supplemental Material that may be linked to, or accessed through, an article. The authors have granted ASM a non-exclusive, world-wide license to publish the Supplemental Material files. Please contact the corresponding author directly for reuse.
