# Supplementary material for: Antimalarial resistance risk in Mozambique detected by a novel quadruplex droplet digital PCR assay
Source: Antimicrob Agents Chemother. 2024 May 21;68(7):e00346-24. doi: 10.1128/aac.00346-24 (PMC11232384; doi:10.1128/aac.00346-24)
Supplement: Supplemental figures — Figures S1 to S11. [file aac.00346-24-s0001.docx]

**Supplemental Figures**

**
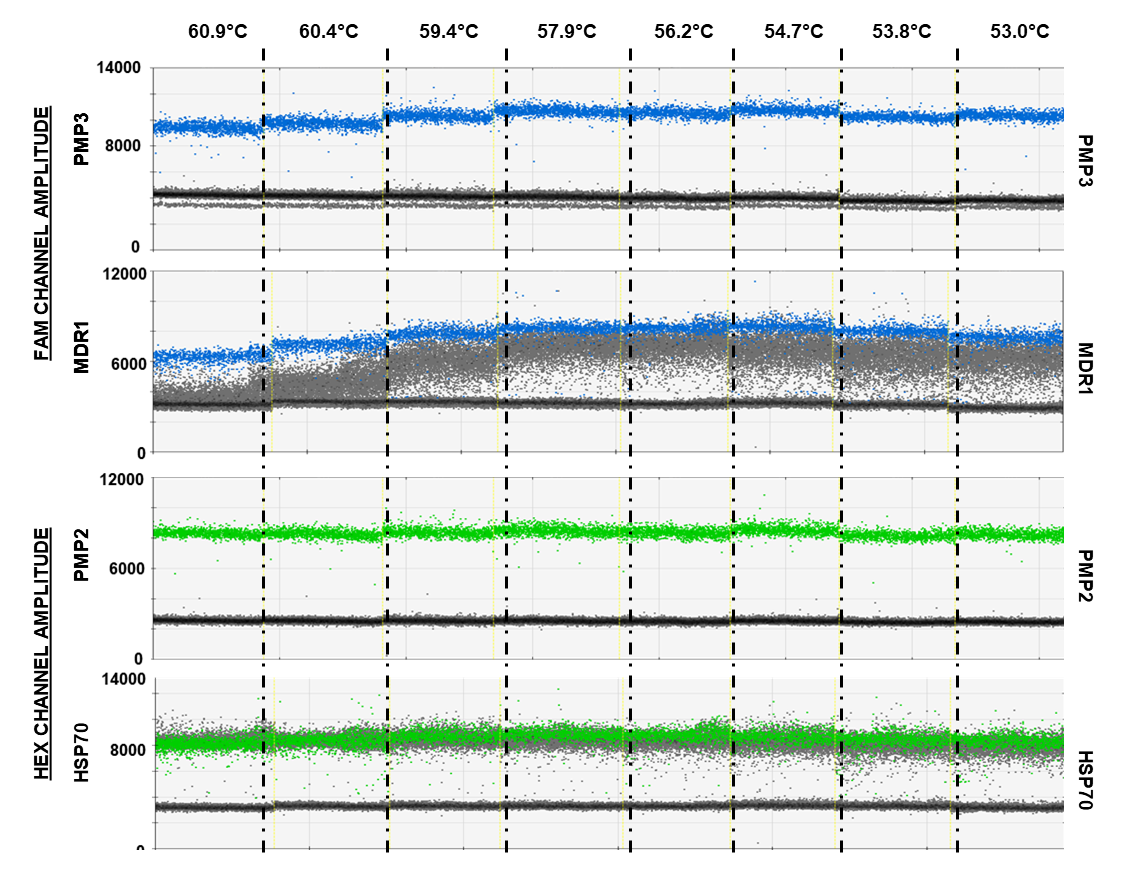
**

**Figure S1:** **Thermal gradient of primer annealing temperature.** Sample: laboratory cultured *3D7* *P. falciparum* DNA, 0.025ng total. Cycling condition: 60 cycles across a thermal gradient, in duplex (*pfpmp2-*HEX with *pfpmp3-*FAM and *pfhsp70-*HEX *with pfmdr1-*FAM). Droplets without assignment (grey) are double positives for that assay.


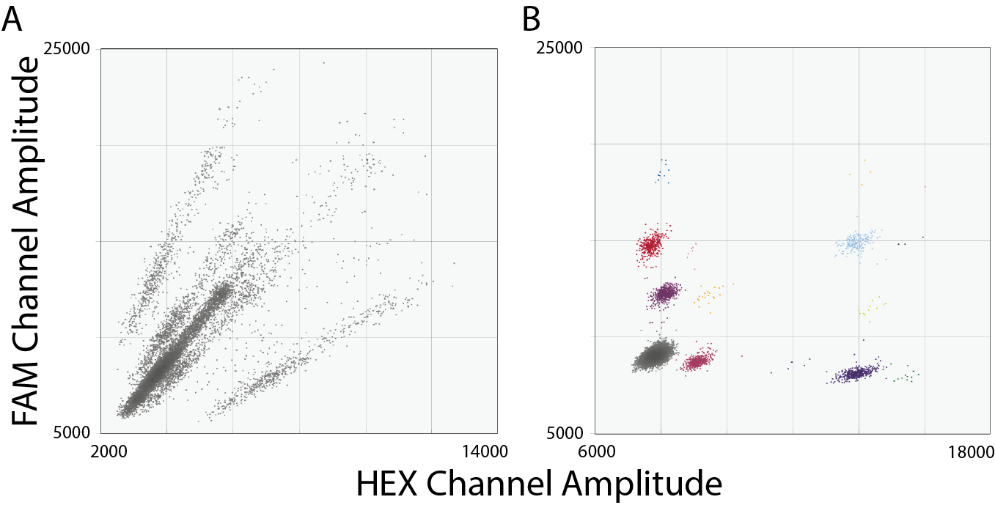


**Figure S2.** **Clinical sample dilution improves quadruplex ddPCR amplification.** Sample: 002A from Maputo, 5μl total. Cycling condition: 60 cycles. **A.** Neat condition (no dilution, 11160 total droplets). **B.** Diluted 1:10 before droplet partitioning and amplification (17358 total droplets). Clusters of droplets are colored as in Figure 1.


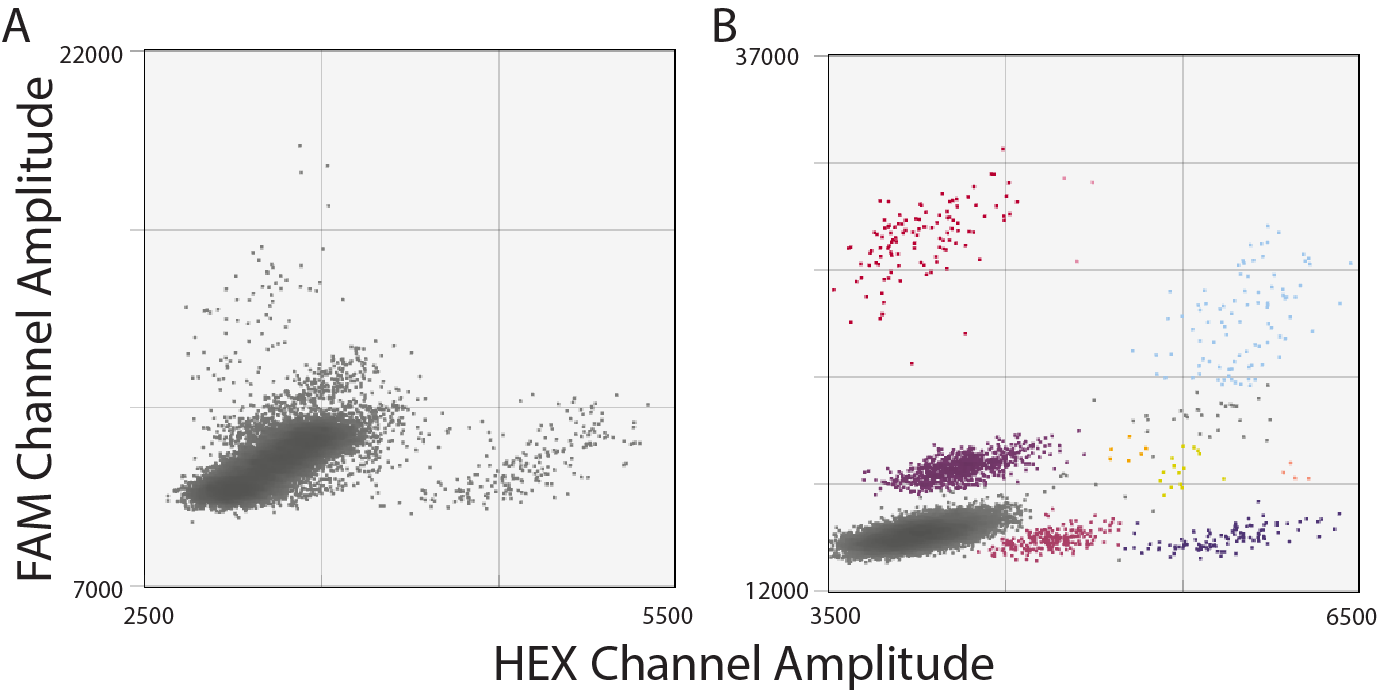


**Figure S3:** **Longer cycling program improves quadruplex ddPCR amplification.** Sample: laboratory cultured *Dd2 P. falciparum* DNA, 0.02ng total. **A.** 40 cycle PCR condition (15631 total droplets). **B.** 60 cycle PCR condition (12751 total droplets). Clusters of droplets are colored as in Figure 1.


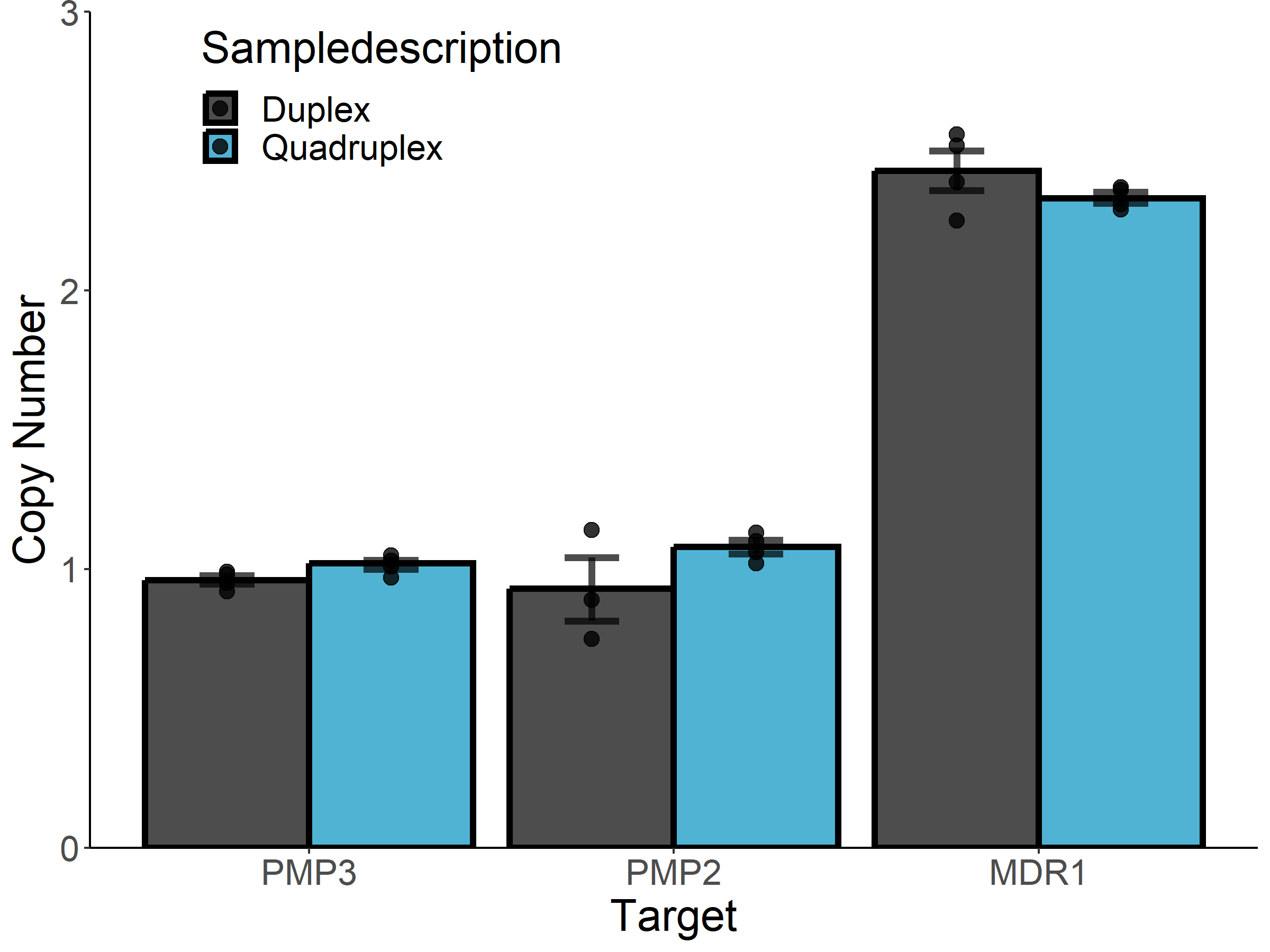


**Figure S4:** **Quadruplex and duplex assays yield consistent copy number quantification.** Sample: *P. falciparum Dd2* genomic DNA, 0.05ng total. Duplex assays used the same primer/probe concentrations as listed for the quadruplex assay and are as follows: *pfpmp3*-FAM/*pfhsp70*-HEX; *pfpmp2*-FAM/*pfhsp70*-HEX; *pfmdr1*-FAM/*pfhsp70*-HEX. Error bars, SEM. Statistical test: student’s unpaired t-test. p= 0.0575; p= 0.1889; p= 0.2430 for *pfpmp3, pfpmp2, and pfmdr1*, respectively.

**
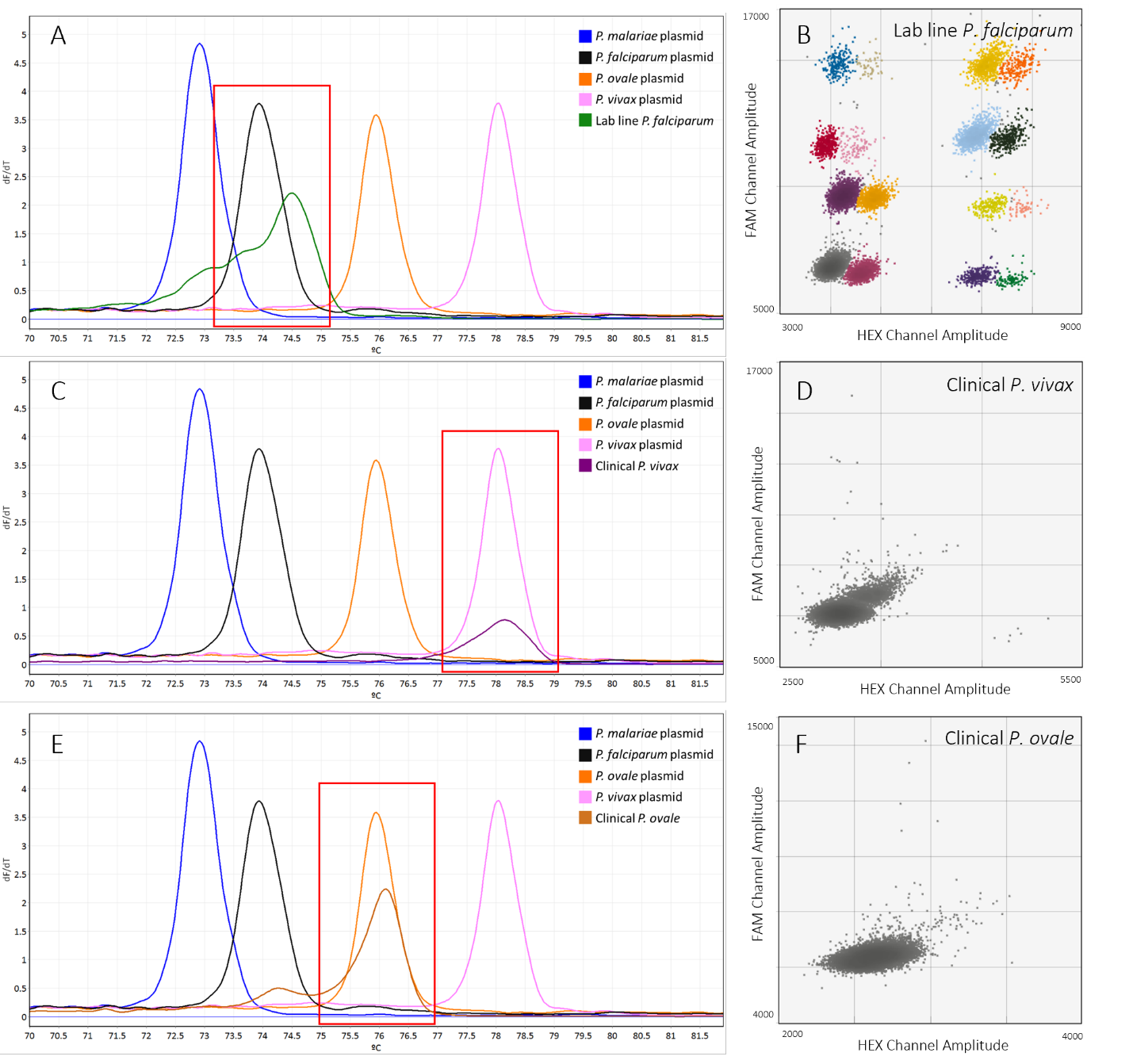
**

**Figure S5. Non-*Plasmodium falciparum*species are not amplified by the quadruplex assay. A, C, E.**Species confirmation using high resolution melt (HRM) analysis of 18s ribosomal RNA genes from various *Plasmodium* species. Control plasmids, which contain single copies of species-specific 18s ribosomal RNA genes, are presented on each graph for comparison to genomic DNA from the test parasite lines or isolates (**A:** Laboratory cultured *Dd2 P. falciparum* genomic DNA; **C:**Genomic DNA from clinical isolate positive for *P. vivax*; and **E:**Genomic DNA from clinical isolate testing for *P. ovale*). Red boxes highlight relevant comparison for each sample. Multiple peaks are diagnostic of genomic DNA samples from specific species due to amplification of distinct 18s rRNA gene copies present in the genomes (# of total 18s rRNA genes in genome/# of 18s rRNA genes with primer homology: *P. falciparum* 5/5; *P. vivax* 3/2; and *P. ovale* *wallikeri* 2/2). **B, D, E.**Representative quadruplex ddPCR performed on each sample, with HRM- confirmed species (**B:***Dd2* *P. falciparum*laboratory line, 15988 total droplets; **D:***P. vivax*clinical sample, 16284 total droplets); and **F:***P. ovale*clinical sample, 13856 total droplets). Grey droplets: droplets containing no target DNA (negative population). Positive droplets colored as in FIG 1.


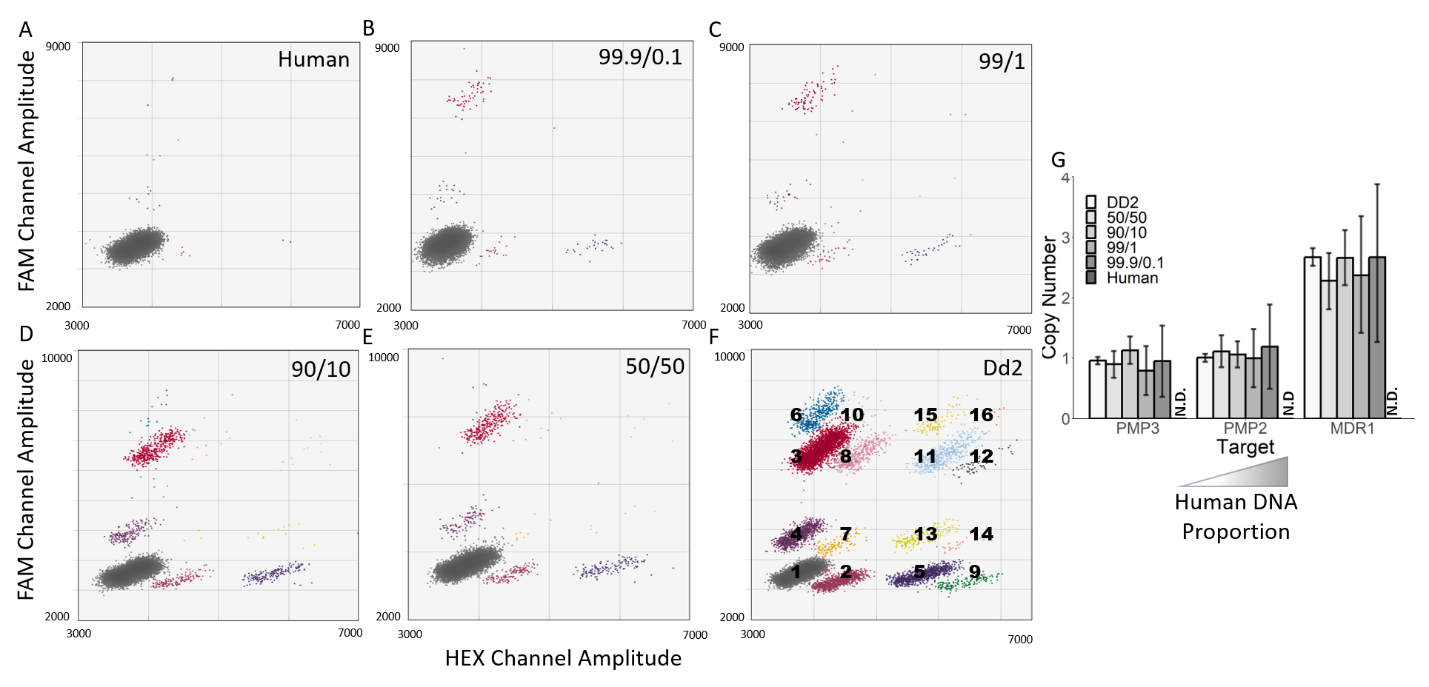


**Figure S6:** **Quadruplex clustering and copy number determination is unaffected by contaminating human DNA.** Sample: laboratory cultured *Dd2 P. falciparum* DNA (*pfmdr1* CNV+) mixed with different proportions of human DNA. **A-F.** Representative ddPCR plots. **A.** 100% Human DNA (input 0.05ng, 15847 total droplets). **B.** 99.9% human DNA (7.92ng) with 0.1% parasite DNA (0.008ng), 17043 total droplets. **C.** 99% human DNA (0.792ng) with 1% parasite DNA (0.008ng), 16837 total droplets. **D.** 90% human DNA (0.792ng) with 10% parasite DNA (0.08ng), 17068 total droplets. **E.** 50% human DNA (0.08ng) with 50% parasite DNA (0.08ng), 14804 total droplets. **F.** 100% *Dd2* *P. falciparum* DNA (0.05ng, 15025 total droplets). (1) droplets containing no target DNA (negative population), (2) droplets containing at least one copy of *pfhsp70*, (3) droplets containing at least one copy of *pfmdr1*, (4) droplets containing at least one copy of *pfpmp3*, (5) droplets containing at least one copy of *pfpmp2*, (6) droplets with both *pfpmp3* and *pfmdr1*, (7) droplets with both *pfmdr1* and *pfhsp70*, (8) droplets with both *pfpmp3* and *pfhsp70*, (9) droplets with both *pfhsp70* and *pfpmp2*, (10) droplets with *pfmdr1*, *pfhsp70*, and *pfpmp3*, (11) droplets with *pfpmp2* and *pfpmp3*, (12) droplets with *pfhsp70*, *pfpmp3*, and *pfpmp2*, (13) droplets with *pfpmp2* and *pfmdr1*, (14) droplets with *pfhsp70*, *pfpmp2*, and *pfmdr1*, (15) droplets with *pfmdr1*, *pfpmp2*, and *pfpmp3*, (16) and droplets with *pfmdr1*, *pfpmp2*, *pfpmp3*, and *pfhsp70* **G.** Determination of copy number for each target throughout experiments performed in A-F. *Dd2* *P. falciparum* is single copy at *pfpmp2* and *pfpmp3* and has 3 copies for *pfmdr1*. N.D., not able to be determined due to too few droplets to quantify. Error bars, 95% CI.


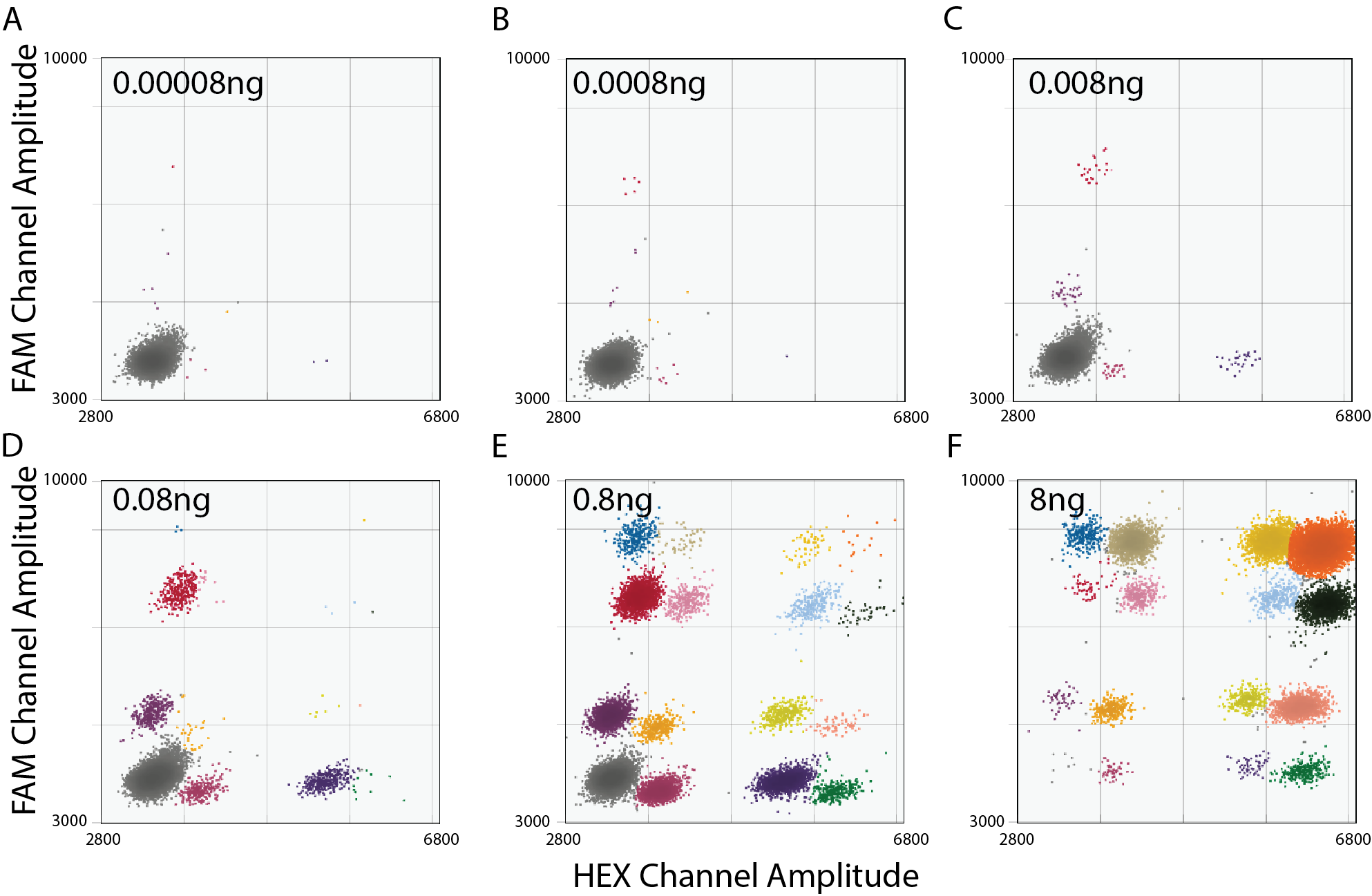


**Figure S7:** **Laboratory parasite DNA dilutions show quadruplex ddPCR assay sensitivity.** Sample: laboratory cultured *NF54 P. falciparum* DNA, 10-fold DNA dilutions. Cycling condition: 60 cycles. Clusters of droplets are colored as in Figure 1. **A.** 0.00008ng, 17349 total droplets. **B.** 0.0008ng, 18266 total droplets. **C.** 0.008ng, 16969 total droplets. **D.** 0.08ng, 17739 total droplets. **E.** 0.8ng, 18262 total droplets. **F.** 8ng, 18180 total droplets.


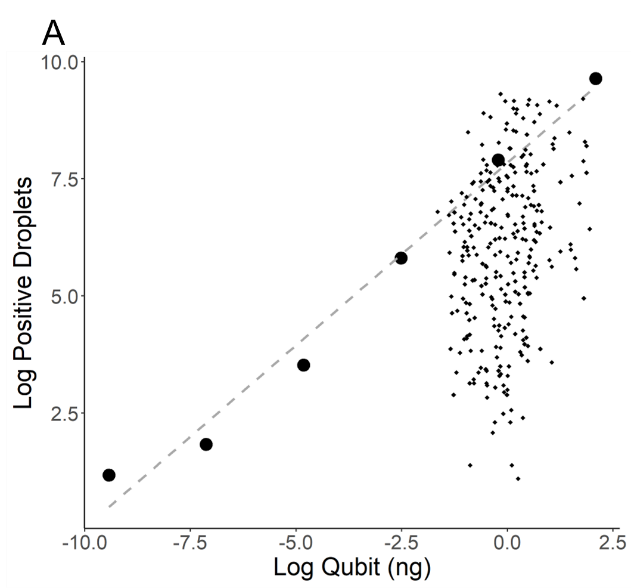

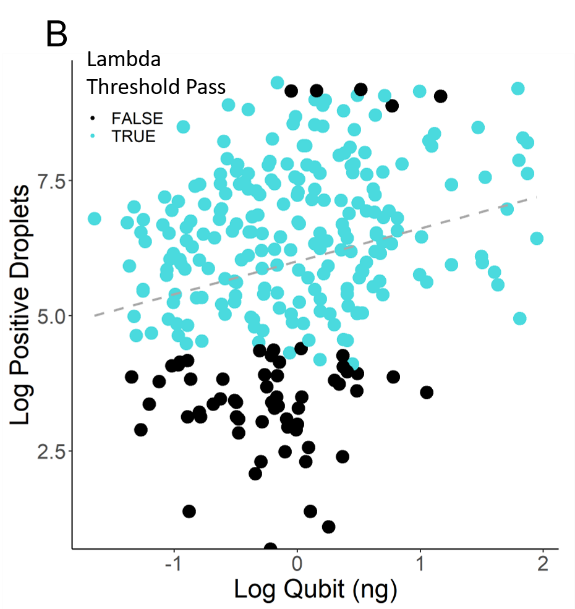


**Figure S8: Diluted parasite DNA displays sensitivity of quadruplex ddPCR assay and clinical sample range.** Quantification of positive droplets by ddPCR compared to DNA concentration using Qubit fluorimeter. Cycling condition: 60 cycles. **A.** Large black circles, 10-fold dilutions of DNA from laboratory cultured *NF54 P. falciparum*, R_2_ = 0.9766, p = 1.3E-4 (0.00008 to 8ng, as in **FIG S7**); small black diamonds, Mozambique clinical samples with measurable DNA concentrations. **B.** Clinical samples with measurable DNA concentrations (n = 283), R_2_ = 0.0599. Blue circles, samples in range of λ (0.005 – 1.1); black circles, samples outside of λ range.

**
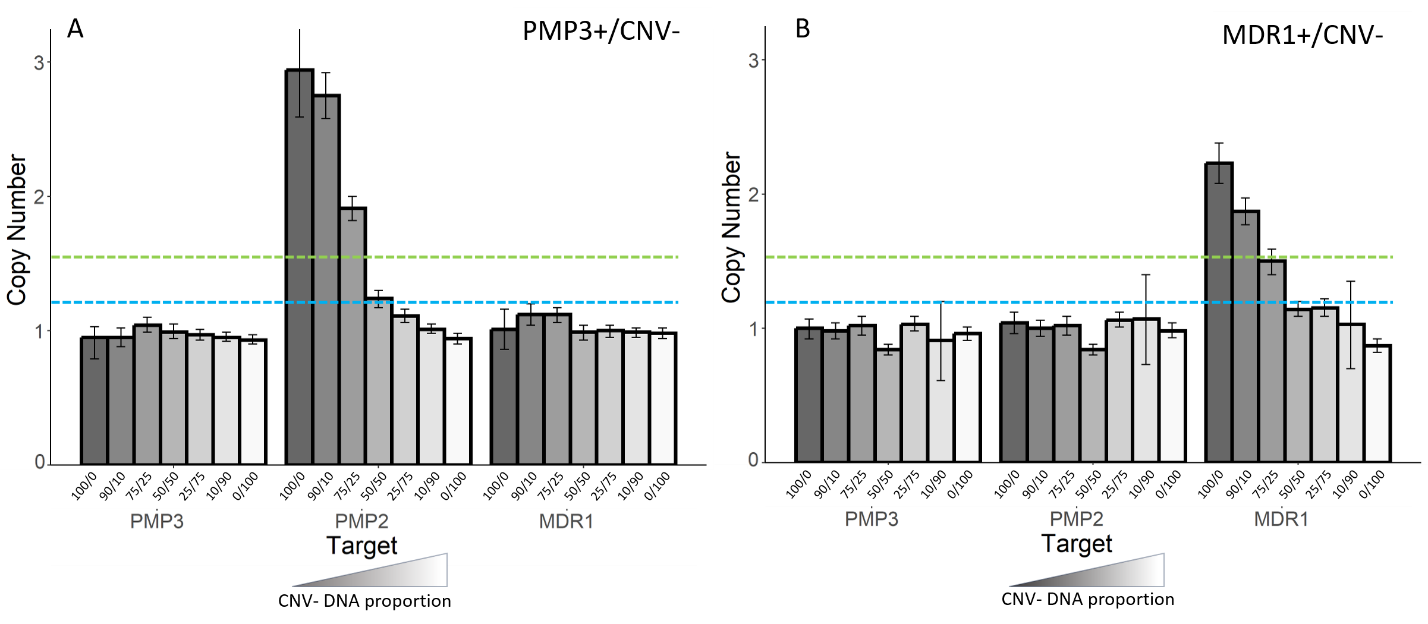
Figure S9:** **Mixed CNV genotypes impact copy number determination in the quadruplex ddPCR assay.** Samples: **A.** PM2GT clone F4 (*pfpmp3* CNV+) mixed with *3D7* (CNV-), 0.05ng total; **B.** *Dd2* (*pfmdr1* CNV+) mixed with *3D7* (CNV-), 0.05ng total. Proportions (CNV+/CNV-) from left to right: 100/0, 90/10, 75/25, 50/50, 25/75, 10/90, and 0/100. Error bars, 95% CI. Blue dotted line: copy number of 1.2; green dotted line: copy number of 1.5.

**
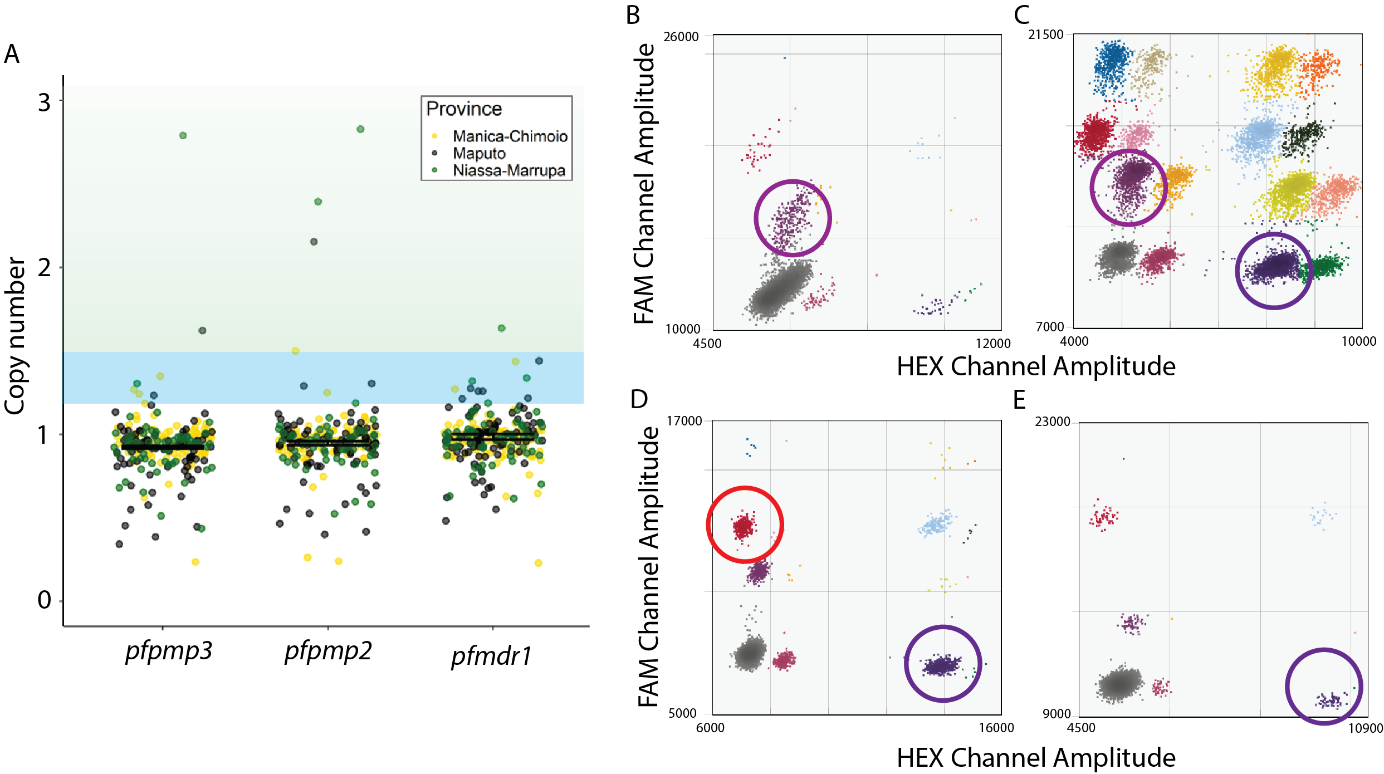
**

**Figure S10**. **Quadruplex ddPCR assay detects CNVs in high quality samples from Mozambique.** **A.** Copy number for each target from high quality clinical samples from the three Mozambique provinces. Error bars, SEM. Blue region; potential CNV samples (copy number of 1.2-1.5), Green region; CNV positive samples (copy number of >1.5). **B-E**. Quadruplex ddPCR assay with clinical sample DNA. All samples were run at 60 cycles at dilutions described in Table S1. Clusters of droplets are colored as in Figure 3. **B.** Sample: 014PC (Manica), *pfmdr1* CNV (copy number =2.6) circled in purple (15083 total droplets). Note: this sample only passed λ= 0.005-1.1 for the *pfmdr1* locus. **C.** Sample: 124PN (Niassa), *pfmdr1* (CN= 1.6) and *pfpmp2* (CN=2.4) CNVs circled in purple and navy, respectively (12104 total droplets). Note: *pfpmp3* CN= 1.3 indicating possible tandem CNV in this sample. **D.** Sample: 012A (Maputo), *pfpmp2* (CN= 2.2) and *pfpmp3* (CN=1.6) CNVs circled in navy and red, respectively (18332 total droplets). **E.** Sample: PC7048 (Manica), *pfpmp2* CNV (CN=1.5) circled in navy (15500 total droplets). Note: *pfmdr1* CN= 1.2 in this sample but it did not pass λ= 0.005-1.1.


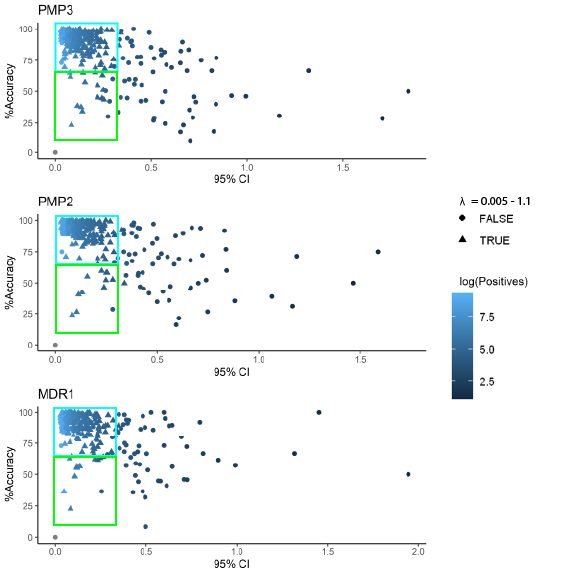


**Figure S11:** **Application of λ threshold identifies high quality samples and predicts samples with potential CNVs**. 95% CI from ddPCR analysis; Accuracy = 100 * |1 – Copy Number| for the *pfpmp3* (top), *pfpmp2* (middle), and *pfmdr1* (bottom) loci. Blue and green boxes indicate high quality samples within a λ range (λ = 0.005-1.1) used for further analysis. Blue heat map indicates the log of positive droplets.
